# Relations of Chemistry to Dentistry

**Published:** 1897-11

**Authors:** J. S. Cassidy

**Affiliations:** Covington, Ky.


### RELATIONS OF CHEMISTRY TO DENTISTRY.[1]

BY J. S. CASSIDY, D.D.S., COVINGTON, KY.

THE subject allotted to me for this occasion is one which I approach with feelings somewhat akin to those of the timid youth who for the first time tells the story of his love, knowing full well that the loved one possesses many attributes of rare perfection inherited from a long line of distinguished ancestors. The heroine of our story is Dentistry, and I take it that her most illustrious progenitor is Chemistry.

Investigation of the laws which were supposed to govern observed natural phenomena was one of the favorite pursuits of learned men from the earliest historic times. Reasoning from effect to cause, as time progressed, led to many useful discoveries, probably in the same ratio to intelligence and the laws of supply and demand as obtains in the affairs of this day and age.

The development of the ancient art of alchemy along practical lines was a leading civilizing influence on the people, as they gradually emerged from their simple surroundings into a clear perception of the comforts which can be obtained from changing material bodies into more desirable or needed conditions. The inhabitants of the desert of Libya were in the olden times inadequately supplied with common salt; their alchemists accordingly manufactured a substitute from camels' dung and named it "sal ammoniac," in honor of Jupiter Ammon, whom they worshipped. Herein the Arab chemists exhibited a commendable commercial spirit, whereas their Greek and Roman contemporaries labored somewhat more in the field of pharmacy by extracting from both mineral and vegetable sources numerous medicinal agents, thus balancing the mercenary by the professional spirit.

Whatever of dentistry existed in those days must have partaken of the benefits derived from the important part played by alchemy in general therapeutics. According to Dr. Ebers, many branches of science flourished wonderfully in ancient Egypt and were practised by specialists of the priestly order. "Our physicians," said the king of Amasia to his visitor, Crœsus, "are only permitted to treat one part of the body. We have aurists, dentists, and oculists, surgeons for fractures of the bone, and others for

---

[1] Read before the American Dental Association at Old Point Comfort, Va., August 4, 1897.

internal diseases." By the ancient priestly law a dentist was not allowed to treat a deaf man, nor a surgeon for broken bones to treat a patient who was suffering from disease of the bowels, even though he had a first-rate knowledge of internal complaints. This law aimed at securing a great deal of real and thorough knowledge,—an aim, indeed, pursued with the most praiseworthy earnestness in all branches of science. "I came to Egypt," wrote the Sybarite, "to consult a dentist. That rude fellow Aristomachus, of Sparta, however, knocked out the defective tooth and so saved me from an operation the thought of which had often made me tremble. On recovering consciousness, I found that three teeth had been knocked into my mouth,—the diseased one and two others which, though healthy, would probably at some future time have caused me pain."

During the long interregnum between the ancient Oriental and the modern European civilization there was such continuous robber warfare and rapine that the arts of peace, outside of the quiet cloister, were not encouraged. There was no dental art worthy of the name, and comparatively little chemical research. In fact, modern chemistry as a science may be said to date its birth from the discovery of oxygen, and coincidently dental science must claim its origin as beginning about the same time. It would be superfluous and beyond the scope of our present purpose to note the advances which these divisions of human effort have made since then. They are both concomitants of higher civilization. "Show me," said Tyndall, "the country where the greatest quantity of sulphuric acid is used, and I will tell you that its people are above all others the most generally enlightened." In like manner we may say that the people of any community where dentistry flourishes are of a higher order of civilization than are those found elsewhere. My own town, by the way, contains its full quota of excellent dentists.

Dentistry is and should be cosmopolitan. It appropriates with due permission facts and methods from the humblest as well as the most exalted occupations, and in return gives back to them a large percentage of the benefits received. Metallurgy, moulding, sculpture, surgery, medicine, physics, and chemistry have been appealed to, not in vain; and as a consequence the accomplished dentist could point out to willing youth the best means of becoming experts in these separate vocations.

Of all these sciences, that of chemistry is the most universal, since it has no limitation; all forms of matter and force must own its sway. In its domain secrecy, mystery, and charlatanism are no longer tolerated, and all who will may partake freely of the benefits it has conferred and will continue to confer in increasing ratio upon mankind.

To chemistry our profession is indebted in that it prepared nitrous oxide, by which means in after years Dr. Horace Wells, a dentist, might be permitted to introduce anæsthesia, the greatest boon ever conferred on suffering humanity. And here let me say that it does not follow that a man must thoroughly understand every department of the noble science in order to appreciate truths in chemistry which may be daily useful to him in our profession. Even a limited knowledge of the principles of chemistry enables the operator to administer that gas intelligently, knowing that nitrous oxide supersedes the normal process of oxidation in the body and prevents the ready elimination of its products, inducing thus the anæsthesia by rapid formation and retention of carbon dioxide, which fact by itself suggests the proper remedies for overcoming the tendency to asphyxia which necessarily follows the inhaling of the gas.

Dentists, as a rule, are not indifferent to nor ignorant of chemical processes, notwithstanding our friend Dr. Crouse asserted that there was not a single dentist in this country who could make an accurate quantitative analysis of any dental amalgam, whereas hundreds might be named who could do so if it were necessary. Indeed, there are many men, not professional chemists, who are able to do excellent work along lines of chemical discovery and manipulation. The recent studies of the chemical and physical properties of amalgams are of inestimable value to every dentist who thankfully takes advantage of the information derived through the labors of Dr. Black.

Moreover, not to mention the innumerable pharmaceutical preparations, our zinc oxide cements may be brought in evidence; and as some of us are sure that fillings of these materials are disposed to disintegrate more at the cervical portion than elsewhere, we also think we know the true and only cause of that unfortunate disposition, in that the part under or near the gum-margin is a favorite point for alkaline fermentation, such as the production of ammonia, resulting in the inevitable abstraction by it of the electro-negative substance of the cement. Some may be inclined to consider this brief allusion to cause and effect as "only a fine-spun theory," but it is not. It is as susceptible of proof as any problem in mathematics. We cannot, as dentists, escape, even if we so desire, the claims of the science of chemistry upon our earnest attention and study, for on every hand we are compelled to admit the insidious influence of its affinities to be the exciting and efficient cause of the principal diseases with which we have to contend. Happily, the study of chemistry, while acquainting us with these potent influences, also points out to us, within the sphere of the same science, the means at hand to combat them most beneficently, provided we will but study and apply them.

No member of this (as the late Atkinson termed it) "Excelsior Association of Dentists" will doubt the culminating discovery of Dr. Miller, that at least lactic acid is developed in the mouth by the presence of bacteria and the materials necessary to their support, and also to the play of chemical affinities at the point of carious destruction, and, further, that the destroying agent acts molecularly with as definite results as pertain to any other natural phenomena.

Some years ago, when the so-called "germ theory" of disease was introduced, there were not a few who received the innovation with satisfaction, because they would not or could not understand, and therefore prove, the chemical theories of Watt. It is now, however, accepted beyond question that there is no conflict between the two theories that bacteria are a necessary factor in fermentation, and that their only trysting-places are in the midst of extraneous material susceptible to that process, the elements of which must obey the laws of their affinities; and if, for instance, among other compounds, destructive and otherwise, lactic acid, as proven by Miller, is developed in contact with a tooth, destruction of that part will proceed molecule by molecule, so that while it is probable that teeth, like other organs, yield to an increased, non-resisting influence of a "periodic law," dental caries is *per se* a disease of purely chemical propagation. From this latter view-point the relations between chemistry and dentistry are not of the most amicable character, notwithstanding which the science whose unseen minions are indirectly responsible for the existence of our principal enemy will inevitably furnish us with more effective weapons of defence than we as yet possess. No wonder that dentists are, perhaps unconsciously, more interested in chemistry than are the members of the mother profession; at least it would so appear by personal contact with them and by the impartial reading of medical and dental journals. One of the former (to refer to a single example) lately expressed itself surprised that any two acids could neutralize each other, and then went on to say that by mixing one part of acetic acid and one and a half of carbolic acid a neutral compound will result. Any tyro in organic chemistry knows that phenol is not an acid, but, on the contrary, is an alcohol, and that an alcohol and an acid combined will produce an ester and water:

$$C_6H_5HO + C_2H_4O_2. \quad C_6H_5C_2H_3O_2 + H_2O.$$

Phenol. Acetic acid. = Phenyl acetate.

Perhaps the worst chemical blunder ever made officially in a dental journal occurred a few years ago in a foreign periodical, wherein the editor, with the characteristic, stupid arrogance of his kind, criticising a brief scientific description of ordinary combustion, wherein light is an essential accompaniment, mentioned the rusting of iron as his beau ideal of that process. We could forgive him for this were it not that he presumed to know it all on all questions of a similar nature.

It goes without saying, that a knowledge of certain rules in chemistry is a great aid in adapting means to ends, when anything new in that line appears, as, for instance, in cataphoresis. We were told at first that the positive pole must be used in contact with whatever drug was employed, whereas it is well known in electrolysis that the radicals or ions of conducting compound liquids separate in a perfectly definite manner, the positive radical being attracted to the cathode and the negative to the anode, so that we will likely obtain better results by selecting anaphoresis when we wish greater penetration of a strongly electro-negative radical. A duly certified list of comparative, positive, and negative classes of radicals has been known for many years, of which we are welcome to take advantage, and in return for all these favors would it not be an act of courtesy on our part to approve, when occasion permits, certain changes in nomenclature, adopted through official international committees appointed for the purpose by the most influential scientific bodies? Our journals, it is almost unnecessary to say, are editorially fully up to and are, indeed, in advance of the times; but we see too often in reports of discussions in our local societies and in some of our latest text-books names of things and terms that shock the sensibilities of euphony and truth. Take, for example, potassium iodide. Is not that name more pleasant to the ear than iodide of potash, especially when we realize that the compound contains no potash whatever? We have been surfeited for a long time with cocaine hydrochlorate, and lately with eucaine hydrochlorate, although a generation ago it was decided that acids like hydrochloric acid (HCl), hydrobromic acid (HBr), etc., whose electro-negative radicals are elementary, confer names on salt which terminate in "ide." The final *e* has been eliminated, therefore the name of every salt of such acids should end in "id," as "cocaine hydrochlorid." It is only those acids which have compound negative radicals that give names to salts ending in "ate" or "ite," as the case may be. Sulphuric acid ($H_2SO_4$) forms sulphates; sulphurous acid ($H_2SO_3$), sulphites; chloric acid ($HClO_3$), chlorates; chlorous acid ($HClO_2$), chlorites, etc.

Life is too short to nominate for consideration at this time more than one other frequently applied word, and that word is "density." We use it, do we not, as a synonyme for hardness in the structural substance of teeth, instead of its true meaning,—*i.e.*, specific gravity? Ice is harder and more compact to a cutting instrument than is liquid water, but it is less dense. The diamond, among the hardest of bodies known, is only three and a half times as heavy as water, while pure gold is comparatively soft.

It is not possible to believe that the specific gravity of a tooth has anything to do with predisposition to disease, or even that compactness of calcium constitutes it a condition that presents a physiological barrier to decay. While perhaps these matters may appear of small practical importance, yet surely, as we claim to be devoted disciples of pure science, we have no right to trifle with accepted nomenclature.

